# Supplementary material for: Modeling glioblastoma heterogeneity as a dynamic network of cell states
Source: Mol Syst Biol. 2021 Sep 16;17(9):e10105. doi: 10.15252/msb.202010105 (PMC8444284; doi:10.15252/msb.202010105)
Supplement: Supplementary file 6 — Source Data for Figure 5 [file MSB-17-e10105-s004.zip › Figure5A_sourcedata/GSEA_3017/nosigdb_stateF.GseaPreranked.1622795015644/RICHARDS_NOWAKOWSKI_SCIENCE_2017_RG_EARLY_UPREG.html]

Details for gene set RICHARDS\_NOWAKOWSKI\_SCIENCE\_2017\_RG\_EARLY\_UPREG[GSEA]

|  || Dataset | state33017 |
| Phenotype | NoPhenotypeAvailable |
| Upregulated in class | na\_neg |
| GeneSet | RICHARDS\_NOWAKOWSKI\_SCIENCE\_2017\_RG\_EARLY\_UPREG |
| Enrichment Score (ES) | -0.5947604 |
| Normalized Enrichment Score (NES) | -4.436067 |
| Nominal p-value | 0.0 |
| FDR q-value | 0.0 |
| FWER p-Value | 0.0 |
Table: GSEA Results Summary

  

Fig 1: Enrichment plot: RICHARDS\_NOWAKOWSKI\_SCIENCE\_2017\_RG\_EARLY\_UPREG      
 Profile of the Running ES Score & Positions of GeneSet Members on the Rank Ordered List

  

| PROBE | GENE SYMBOL | GENE\_TITLE | RANK IN GENE LIST | RANK METRIC SCORE | RUNNING ES | CORE ENRICHMENT || 1 | FTH1 |  |  | 19 | 1.203 | -0.0006 | No |
| 2 | PNRC1 |  |  | 23 | 1.070 | 0.0056 | No |
| 3 | RPS27L |  |  | 33 | 1.025 | 0.0086 | No |
| 4 | CMBL |  |  | 49 | 0.935 | 0.0080 | No |
| 5 | COL4A5 |  |  | 71 | 0.829 | 0.0037 | No |
| 6 | PON2 |  |  | 93 | 0.764 | -0.0011 | No |
| 7 | PEG3 |  |  | 162 | 0.645 | -0.0297 | No |
| 8 | ATP1B1 |  |  | 167 | 0.635 | -0.0271 | No |
| 9 | C1orf61 |  |  | 202 | 0.587 | -0.0395 | No |
| 10 | TRIAP1 |  |  | 208 | 0.584 | -0.0378 | No |
| 11 | HSPA4L |  |  | 215 | 0.579 | -0.0365 | No |
| 12 | LIFR |  |  | 239 | 0.562 | -0.0438 | No |
| 13 | PIK3R3 |  |  | 251 | 0.553 | -0.0452 | No |
| 14 | FAM162A |  |  | 282 | 0.524 | -0.0561 | No |
| 15 | ATP6V0E1 |  |  | 303 | 0.510 | -0.0622 | No |
| 16 | HES1 |  |  | 320 | 0.501 | -0.0664 | No |
| 17 | SEMA5A |  |  | 336 | 0.494 | -0.0702 | No |
| 18 | PDCD4 |  |  | 360 | 0.484 | -0.0780 | No |
| 19 | RPS19 |  |  | 397 | 0.471 | -0.0922 | No |
| 20 | MAGED2 |  |  | 405 | 0.467 | -0.0923 | No |
| 21 | SLC3A2 |  |  | 411 | 0.466 | -0.0913 | No |
| 22 | EEF1A1 |  |  | 417 | 0.464 | -0.0905 | No |
| 23 | DDIT3 |  |  | 447 | 0.451 | -0.1014 | No |
| 24 | TTYH1 |  |  | 457 | 0.448 | -0.1026 | No |
| 25 | BTBD1 |  |  | 459 | 0.447 | -0.0998 | No |
| 26 | TIPARP |  |  | 472 | 0.444 | -0.1025 | No |
| 27 | HEY1 |  |  | 496 | 0.436 | -0.1106 | No |
| 28 | YBX3 |  |  | 509 | 0.431 | -0.1134 | No |
| 29 | FGFR3 |  |  | 515 | 0.429 | -0.1128 | No |
| 30 | PEA15 |  |  | 521 | 0.427 | -0.1121 | No |
| 31 | CNN3 |  |  | 576 | 0.407 | -0.1356 | No |
| 32 | HDAC9 |  |  | 579 | 0.404 | -0.1337 | No |
| 33 | IER5 |  |  | 605 | 0.396 | -0.1431 | No |
| 34 | ID4 |  |  | 659 | 0.383 | -0.1663 | No |
| 35 | GABARAP |  |  | 746 | 0.359 | -0.2058 | No |
| 36 | MASP1 |  |  | 755 | 0.357 | -0.2071 | No |
| 37 | MAP1B |  |  | 762 | 0.355 | -0.2075 | No |
| 38 | TPT1 |  |  | 786 | 0.350 | -0.2162 | No |
| 39 | TSPAN6 |  |  | 838 | 0.340 | -0.2388 | No |
| 40 | VEGFA |  |  | 840 | 0.339 | -0.2368 | No |
| 41 | ZFP36L2 |  |  | 850 | 0.336 | -0.2388 | No |
| 42 | PPM1D |  |  | 856 | 0.334 | -0.2388 | No |
| 43 | ZFHX4 |  |  | 878 | 0.330 | -0.2467 | No |
| 44 | SLC5A3 |  |  | 957 | 0.316 | -0.2826 | No |
| 45 | FAT1 |  |  | 968 | 0.315 | -0.2853 | No |
| 46 | ITGB8 |  |  | 970 | 0.314 | -0.2835 | No |
| 47 | MSI1 |  |  | 1051 | 0.300 | -0.3205 | No |
| 48 | OTX1 |  |  | 1102 | 0.293 | -0.3428 | No |
| 49 | SDK2 |  |  | 1146 | 0.286 | -0.3618 | No |
| 50 | EFNB1 |  |  | 1152 | 0.285 | -0.3622 | No |
| 51 | CKB |  |  | 1200 | 0.279 | -0.3832 | No |
| 52 | IRF2BP2 |  |  | 1311 | 0.267 | -0.4351 | No |
| 53 | RFX4 |  |  | 1320 | 0.266 | -0.4371 | No |
| 54 | ZDHHC2 |  |  | 1342 | 0.262 | -0.4455 | No |
| 55 | GAPDH |  |  | 1361 | 0.261 | -0.4525 | No |
| 56 | ABHD3 |  |  | 1382 | 0.259 | -0.4604 | No |
| 57 | EAF1 |  |  | 1440 | 0.254 | -0.4865 | No |
| 58 | PTAR1 |  |  | 1441 | 0.254 | -0.4846 | No |
| 59 | SNRPG |  |  | 1486 | -0.254 | -0.5044 | No |
| 60 | DCLRE1C |  |  | 1493 | -0.257 | -0.5054 | No |
| 61 | HSPH1 |  |  | 1495 | -0.258 | -0.5041 | No |
| 62 | CBFB |  |  | 1499 | -0.259 | -0.5037 | No |
| 63 | SAR1A |  |  | 1505 | -0.262 | -0.5042 | No |
| 64 | RBBP8 |  |  | 1518 | -0.265 | -0.5082 | No |
| 65 | HSP90AA1 |  |  | 1535 | -0.268 | -0.5141 | No |
| 66 | NPM1 |  |  | 1543 | -0.270 | -0.5156 | No |
| 67 | RNPS1 |  |  | 1548 | -0.271 | -0.5156 | No |
| 68 | CREB5 |  |  | 1561 | -0.275 | -0.5195 | No |
| 69 | PSIP1 |  |  | 1564 | -0.275 | -0.5185 | No |
| 70 | IQGAP3 |  |  | 1584 | -0.282 | -0.5257 | No |
| 71 | ZFP36L1 |  |  | 1590 | -0.284 | -0.5261 | No |
| 72 | RPS24 |  |  | 1608 | -0.287 | -0.5324 | No |
| 73 | PSMB7 |  |  | 1611 | -0.288 | -0.5313 | No |
| 74 | TMEM97 |  |  | 1629 | -0.293 | -0.5375 | No |
| 75 | RPL8 |  |  | 1647 | -0.299 | -0.5437 | No |
| 76 | IGF2BP2 |  |  | 1658 | -0.304 | -0.5464 | No |
| 77 | ENO1 |  |  | 1673 | -0.308 | -0.5510 | No |
| 78 | H1FX |  |  | 1705 | -0.321 | -0.5639 | No |
| 79 | PHLDA1 |  |  | 1718 | -0.324 | -0.5674 | No |
| 80 | NFYB |  |  | 1721 | -0.326 | -0.5661 | No |
| 81 | CDC25C |  |  | 1724 | -0.327 | -0.5647 | No |
| 82 | TCF3 |  |  | 1731 | -0.331 | -0.5652 | No |
| 83 | MCM3 |  |  | 1738 | -0.335 | -0.5658 | No |
| 84 | HSPD1 |  |  | 1742 | -0.336 | -0.5648 | No |
| 85 | GLO1 |  |  | 1755 | -0.342 | -0.5682 | No |
| 86 | PTBP1 |  |  | 1756 | -0.342 | -0.5658 | No |
| 87 | RPS26 |  |  | 1767 | -0.347 | -0.5681 | No |
| 88 | RPS2 |  |  | 1770 | -0.348 | -0.5666 | No |
| 89 | RAN |  |  | 1772 | -0.350 | -0.5646 | No |
| 90 | RBM8A |  |  | 1777 | -0.351 | -0.5640 | No |
| 91 | TOMM5 |  |  | 1818 | -0.374 | -0.5809 | No |
| 92 | RPL35 |  |  | 1820 | -0.375 | -0.5787 | No |
| 93 | NUP50 |  |  | 1823 | -0.376 | -0.5770 | No |
| 94 | LDHA |  |  | 1833 | -0.380 | -0.5786 | No |
| 95 | NOP58 |  |  | 1834 | -0.381 | -0.5759 | No |
| 96 | BARD1 |  |  | 1839 | -0.384 | -0.5751 | No |
| 97 | CCT4 |  |  | 1840 | -0.384 | -0.5723 | No |
| 98 | SVIP |  |  | 1849 | -0.388 | -0.5734 | No |
| 99 | RPF2 |  |  | 1853 | -0.391 | -0.5721 | No |
| 100 | CEP135 |  |  | 1855 | -0.393 | -0.5697 | No |
| 101 | TCP1 |  |  | 1875 | -0.401 | -0.5762 | No |
| 102 | RPL12 |  |  | 1895 | -0.418 | -0.5824 | No |
| 103 | HMGA1 |  |  | 1915 | -0.433 | -0.5886 | No |
| 104 | WDR43 |  |  | 1920 | -0.438 | -0.5874 | No |
| 105 | UHRF1 |  |  | 1921 | -0.438 | -0.5843 | No |
| 106 | SNRPB |  |  | 1937 | -0.447 | -0.5884 | No |
| 107 | NAP1L1 |  |  | 1951 | -0.455 | -0.5915 | Yes |
| 108 | DDX39A |  |  | 1957 | -0.458 | -0.5906 | Yes |
| 109 | HSP90AB1 |  |  | 1963 | -0.461 | -0.5898 | Yes |
| 110 | PTGES3 |  |  | 1970 | -0.467 | -0.5893 | Yes |
| 111 | SNHG1 |  |  | 1973 | -0.469 | -0.5869 | Yes |
| 112 | EIF3J |  |  | 1976 | -0.471 | -0.5845 | Yes |
| 113 | TIMELESS |  |  | 1989 | -0.482 | -0.5869 | Yes |
| 114 | MCM4 |  |  | 1993 | -0.487 | -0.5849 | Yes |
| 115 | SPAG5 |  |  | 1997 | -0.490 | -0.5828 | Yes |
| 116 | XPO1 |  |  | 2007 | -0.500 | -0.5836 | Yes |
| 117 | CHEK1 |  |  | 2013 | -0.504 | -0.5824 | Yes |
| 118 | SIVA1 |  |  | 2014 | -0.505 | -0.5788 | Yes |
| 119 | HAUS8 |  |  | 2026 | -0.517 | -0.5805 | Yes |
| 120 | CACYBP |  |  | 2028 | -0.518 | -0.5772 | Yes |
| 121 | HELLS |  |  | 2038 | -0.528 | -0.5778 | Yes |
| 122 | SNRPA1 |  |  | 2040 | -0.528 | -0.5745 | Yes |
| 123 | CDCA3 |  |  | 2041 | -0.529 | -0.5707 | Yes |
| 124 | TTK |  |  | 2047 | -0.533 | -0.5693 | Yes |
| 125 | PPA1 |  |  | 2049 | -0.534 | -0.5659 | Yes |
| 126 | KIF22 |  |  | 2053 | -0.541 | -0.5635 | Yes |
| 127 | CTNNAL1 |  |  | 2059 | -0.548 | -0.5620 | Yes |
| 128 | SNRPE |  |  | 2062 | -0.565 | -0.5589 | Yes |
| 129 | FANCD2 |  |  | 2068 | -0.577 | -0.5572 | Yes |
| 130 | ZWINT |  |  | 2069 | -0.577 | -0.5531 | Yes |
| 131 | CEP152 |  |  | 2070 | -0.578 | -0.5489 | Yes |
| 132 | RTKN2 |  |  | 2071 | -0.579 | -0.5447 | Yes |
| 133 | RACGAP1 |  |  | 2072 | -0.579 | -0.5406 | Yes |
| 134 | MND1 |  |  | 2074 | -0.586 | -0.5368 | Yes |
| 135 | LSM4 |  |  | 2076 | -0.587 | -0.5331 | Yes |
| 136 | SRSF3 |  |  | 2081 | -0.606 | -0.5307 | Yes |
| 137 | CENPK |  |  | 2082 | -0.607 | -0.5263 | Yes |
| 138 | USP1 |  |  | 2084 | -0.612 | -0.5224 | Yes |
| 139 | KNSTRN |  |  | 2085 | -0.615 | -0.5180 | Yes |
| 140 | KIF18A |  |  | 2093 | -0.636 | -0.5168 | Yes |
| 141 | ITGB3BP |  |  | 2102 | -0.652 | -0.5160 | Yes |
| 142 | SNRPD1 |  |  | 2105 | -0.653 | -0.5123 | Yes |
| 143 | CKAP2L |  |  | 2109 | -0.663 | -0.5090 | Yes |
| 144 | CKS2 |  |  | 2115 | -0.694 | -0.5064 | Yes |
| 145 | RAD51AP1 |  |  | 2127 | -0.715 | -0.5067 | Yes |
| 146 | ODC1 |  |  | 2128 | -0.718 | -0.5015 | Yes |
| 147 | CKAP2 |  |  | 2131 | -0.729 | -0.4972 | Yes |
| 148 | KIF2C |  |  | 2136 | -0.756 | -0.4937 | Yes |
| 149 | KIF5B |  |  | 2137 | -0.756 | -0.4883 | Yes |
| 150 | BRCA1 |  |  | 2138 | -0.758 | -0.4828 | Yes |
| 151 | KIF14 |  |  | 2141 | -0.773 | -0.4782 | Yes |
| 152 | NDC80 |  |  | 2142 | -0.775 | -0.4727 | Yes |
| 153 | KIF23 |  |  | 2145 | -0.782 | -0.4680 | Yes |
| 154 | GMNN |  |  | 2146 | -0.785 | -0.4624 | Yes |
| 155 | ANLN |  |  | 2149 | -0.797 | -0.4576 | Yes |
| 156 | DEK |  |  | 2154 | -0.817 | -0.4537 | Yes |
| 157 | DUT |  |  | 2155 | -0.824 | -0.4477 | Yes |
| 158 | NCAPG2 |  |  | 2156 | -0.827 | -0.4418 | Yes |
| 159 | ECT2 |  |  | 2163 | -0.846 | -0.4386 | Yes |
| 160 | HMGB3 |  |  | 2164 | -0.852 | -0.4325 | Yes |
| 161 | KIF20B |  |  | 2165 | -0.852 | -0.4263 | Yes |
| 162 | SPC24 |  |  | 2166 | -0.853 | -0.4202 | Yes |
| 163 | ARHGAP11A |  |  | 2167 | -0.855 | -0.4141 | Yes |
| 164 | BUB1B |  |  | 2174 | -0.871 | -0.4107 | Yes |
| 165 | CCNA2 |  |  | 2181 | -0.902 | -0.4072 | Yes |
| 166 | MIS18BP1 |  |  | 2183 | -0.903 | -0.4011 | Yes |
| 167 | KIF15 |  |  | 2185 | -0.915 | -0.3950 | Yes |
| 168 | KIF4A |  |  | 2186 | -0.921 | -0.3884 | Yes |
| 169 | PRR11 |  |  | 2187 | -0.930 | -0.3817 | Yes |
| 170 | CENPW |  |  | 2192 | -0.943 | -0.3769 | Yes |
| 171 | UBE2T |  |  | 2196 | -0.965 | -0.3714 | Yes |
| 172 | CDKN3 |  |  | 2199 | -0.979 | -0.3653 | Yes |
| 173 | CCNB2 |  |  | 2200 | -0.983 | -0.3582 | Yes |
| 174 | CKS1B |  |  | 2201 | -0.987 | -0.3511 | Yes |
| 175 | HIST1H1E |  |  | 2202 | -0.992 | -0.3440 | Yes |
| 176 | SMC2 |  |  | 2205 | -1.006 | -0.3377 | Yes |
| 177 | KIF11 |  |  | 2206 | -1.008 | -0.3305 | Yes |
| 178 | EZH2 |  |  | 2207 | -1.017 | -0.3231 | Yes |
| 179 | SPC25 |  |  | 2208 | -1.019 | -0.3158 | Yes |
| 180 | CENPE |  |  | 2211 | -1.036 | -0.3093 | Yes |
| 181 | CENPN |  |  | 2217 | -1.079 | -0.3040 | Yes |
| 182 | UBE2C |  |  | 2218 | -1.081 | -0.2962 | Yes |
| 183 | CENPU |  |  | 2219 | -1.088 | -0.2884 | Yes |
| 184 | H2AFZ |  |  | 2220 | -1.090 | -0.2805 | Yes |
| 185 | HMGB1 |  |  | 2221 | -1.116 | -0.2725 | Yes |
| 186 | NCAPG |  |  | 2222 | -1.122 | -0.2644 | Yes |
| 187 | CCNB1 |  |  | 2224 | -1.148 | -0.2566 | Yes |
| 188 | CDK1 |  |  | 2225 | -1.153 | -0.2483 | Yes |
| 189 | PTTG1 |  |  | 2226 | -1.198 | -0.2397 | Yes |
| 190 | ANP32E |  |  | 2227 | -1.200 | -0.2310 | Yes |
| 191 | MAD2L1 |  |  | 2228 | -1.204 | -0.2223 | Yes |
| 192 | DBF4 |  |  | 2232 | -1.247 | -0.2148 | Yes |
| 193 | HMGN2 |  |  | 2233 | -1.267 | -0.2057 | Yes |
| 194 | RRM2 |  |  | 2234 | -1.268 | -0.1966 | Yes |
| 195 | DLGAP5 |  |  | 2235 | -1.273 | -0.1874 | Yes |
| 196 | SMC4 |  |  | 2236 | -1.281 | -0.1782 | Yes |
| 197 | PBK |  |  | 2237 | -1.325 | -0.1686 | Yes |
| 198 | ATAD2 |  |  | 2238 | -1.381 | -0.1587 | Yes |
| 199 | PRC1 |  |  | 2240 | -1.397 | -0.1491 | Yes |
| 200 | HMGB2 |  |  | 2241 | -1.469 | -0.1385 | Yes |
| 201 | BIRC5 |  |  | 2242 | -1.469 | -0.1279 | Yes |
| 202 | HIST1H1C |  |  | 2243 | -1.485 | -0.1172 | Yes |
| 203 | ASPM |  |  | 2244 | -1.490 | -0.1065 | Yes |
| 204 | TOP2A |  |  | 2246 | -1.562 | -0.0958 | Yes |
| 205 | NUSAP1 |  |  | 2247 | -1.600 | -0.0842 | Yes |
| 206 | TPX2 |  |  | 2248 | -1.609 | -0.0726 | Yes |
| 207 | CENPF |  |  | 2249 | -1.691 | -0.0605 | Yes |
| 208 | HIST1H1D |  |  | 2250 | -1.720 | -0.0481 | Yes |
| 209 | HIST1H1B |  |  | 2251 | -1.796 | -0.0351 | Yes |
| 210 | MKI67 |  |  | 2252 | -1.892 | -0.0215 | Yes |
| 211 | HIST1H4C |  |  | 2253 | -2.984 | 0.0000 | Yes |
Table: GSEA details [plain text format]

  

Fig 2: RICHARDS\_NOWAKOWSKI\_SCIENCE\_2017\_RG\_EARLY\_UPREG: Random ES distribution      
 Gene set null distribution of ES for **RICHARDS\_NOWAKOWSKI\_SCIENCE\_2017\_RG\_EARLY\_UPREG**
